# The Relative Contribution of Dietary Habits, Leisure-Time Exercise, Exercise Attitude, and Body Mass Index to Self-Rated Health among College Students in Taiwan

**DOI:** 10.3390/ijerph15050967

**Published:** 2018-05-11

**Authors:** Huey-Hong Hsieh, Chia-Ming Chang, Li-Wei Liu, Hsiu-Chin Huang

**Affiliations:** 1 Department of Leisure Management, Taiwan Shoufu University, Tainan 72153, Taiwan; 2 Department of Physical Education, Health & Recreation, National Chiayi University, Chiayi 60004, Taiwan; gr5166@yahoo.com.tw; 3 Department of Leisure Service Management, Chaoyang University of Technology, Taichung 41349, Taiwan; ijofsrm@gmail.com; 4 Department of Tourism, Leisure, Entertainment Management, Tatung Institute of Technology, Chiayi 60044, Taiwan; op5166@yahoo.com.tw

**Keywords:** dietary habits, college students, leisure-time exercise, exercise attitude, body mass index, self-rated health

## Abstract

*Background*: Self-rated health (SRH) is consistent with objective health status and can serve as a global measure of health status in the general population. The purpose of this study is to find the connections of dietary habits, leisure-time exercise, exercise attitude, and body mass index (BMI) to SRH among college students. *Methods*: The “dietary–exercise attitude and SRH” questionnaire was developed to investigate college students in Taiwan through the Internet. Partial least squares structural equation modeling (PLS-SEM) was used to test the relationship among them. *Results*: The reliability and validity were confirmed using PLS-SEM. The results found exercise habits, dietary habits, and BMI explained 26.5% of SRH. Poor dietary habits and being overweight led to bad health status (negative path coefficients to SRH). Additionally, the study found that positive exercise attitude had a positive relationship with exercise habits. *Conclusions*: Based on the results, college students should be well-informed of the potential threat of poor dietary habits and being overweight to health and should improve their attitude with respect to exercise so as to prevent overweight-related diseases.

## 1. Introduction

With the prevalence of communication products, robots, sedentary lifestyles, and poor dietary habits, obesity is common among all age groups more than ever. Obesity is a major public health problem due to its increasing prevalence [1] and its associations with higher morbidity and mortality from multiple diseases [2,3].

In Chinese society, the emphasis on academic success is well known around the world. Most students only need to study hard and parents do the rest [4]. The focus of study causes the neglect of knowledge of healthy lifestyles, which in turn may lead to health problems. Upon coming to college, they need to take care of themselves. Studies [5,6] of the changes of diet and exercise in Taiwan have shown the trends of poor dietary habits (e.g., consuming sugary drinks) and the decrease of leisure-time exercise. Without the parents’ supervision of diet and lifestyle, being overweight or obese has become prevalent among college students in Taiwan [6].

Research has shown that self-rated health (SRH) is a good indicator of health [7]. In addition, Singh-Manoux et al. [8] found that the measure of physical and mental health contributes most to the SRH construct.

For many people, college is the last education before a career. Therefore, it is very important to cultivate good dietary and exercise habits during this period. The founder of the world's first and largest silicon foundry, Morris Chang, gave 11 admonitions to college students. The top one advises students “to develop a lifetime, healthy lifestyle” [9].

To better know the current status of students’ perceptions of dietary habits, exercise habits, body mass index (BMI), and SRH, this study conducted a series of investigations on college students to identify the relationships of dietary habits, exercise attitude, exercise habits, BMI, and SRH among college students. The results potentially provide useful information for college students and administrators. Therefore, the study intended to answer the following questions: (1) Are the health behavior variables directly related to SRH? (2) Are the exercise attitude variables directly related to exercise habits? (3) Are the health behavior variables directly related to BMI? (4) Are the exercise habit variables directly related to BMI? (5) Is BMI directly related to SRH? (6) Does BMI mediate the relation of health behavior to SRH? (7) Do exercise habits mediate the relation of exercise attitude to SRH?

### 1.1. Self-Rated Health

SRH, a subjective assessment of health status, is extensively used in the public health field. Research had found relevance of SRH and objective health status, and SRH is consistent with objective health status. Therefore, it can serve as a global measure of health status in the general population [9].

### 1.2. Dietary Habits among College Students

Poor eating habits are an important public health issue that has large health and economic implications. Many food preferences are established early, but because people make independent eating decisions more often as they move through adolescence, the transition to independent living during university is important [10,11]. We therefore investigated college students’ dietary habits and examine the connections of the habits to BMI and SRH.

### 1.3. Exercise Attitude and Exercise Habits

Many studies have examined the connection between exercise belief and exercise habits [12]. Several studies have found that positive exercise attitude contributed to the maintenance of good exercise habits [13]. We further examined the relationships of exercise attitude to exercise habits and exercise habits to BMI and SRH.

### 1.4. BMI and Self-Rated Health

BMI is one of the most commonly used measures of obesity. The link between BMI and health shows that overweight or obese people are more likely than those at normal weight to have medical problems such as high blood pressure, diabetes, stroke, and cancer [14]. As Calle et al. [15] pointed out, the risk of death from all causes—cardiovascular disease, cancer, or other diseases—increases throughout the range of moderate and severe overweight conditions for both men and women in all age groups. Since BMI is used as a convenient index for health status, we will examine the link between BMI and SRH.

## 2. Materials and Methods

### 2.1. Participants

The data for this study came from college students in Taiwan. We post a web-based questionnaire and used snowball sampling to invite college students to participate in the study. Two hundred seventy participants completed the investigation.

Since BMI is a key independent variable in the study, students who had not answered the self-reported height and weight measurements were excluded from the study. Therefore, 238 remained in the study, with 121 (50.8%) male and 117 (49.2%) female students.

### 2.2. Measures

Exercise Attitude (EA). The “Exercise Attitude for College Students Scale” developed by Yang & Ku [16] was used to measure respondents’ exercise attitude on a five-point Likert scale (1 = “strongly disagree” to 5 = “strongly agree”). One item asked respondents whether they agree with “Exercise is good for health”. Reliability of the scale is acceptable with a Cronbach’s α of 0.83.

Exercise Habits (EH). The engagement of exercise can be accessed by one’s “frequency”, ”duration”, and “intensity” of exercise involvement in a certain period [17]. In this study, college students’ exercise habits were measured by “frequency”, ”duration”, “intensity”, and “seriousness” of their exercise habits on a five-point Likert scale (1 = “never” to 5 = “always”). In one item, students were asked if they do exercise at least 3 times and 30 min a week.

BMI. BMI was measured by asking respondents to self-report their weight and height. BMI was calculated using the weight (in kilograms) divided by the height squared (in meters).

Dietary Habits. The “Dietary Habits Scale” developed by Huang [18] was used to measure the dietary habits with Cronbach’s α of 0.85. There were seven good dietary habits (GDHs) on the scale. To measure if respondents consumed a good diet, we used the variable labeled “good diet”. One item was to ask respondents to self-report how often in one week they ate “vegetables”. The response format was 1 = almost never, 2 = less than once a week, 3 = every week, 4 = once a day, and 5 = more than once a day. There were nine poor dietary habits (PDH) on the scale. To measure if respondents consumed a poor diet, we used the variable labeled “poor diet”. One item was to ask respondents self-report how often in one week they ate “fast food, e.g., hamburger, fries, hot dog”. The response format was 1 = almost never, 2 = less than once a week, 3 = every week, 4 = once a day, and 5 = more than once a day.

Self-rated Health (SRH). We used two items to measure self-rated health on a 5-point Likert scale (“poor”, “fair”, “good”, “very good”, “excellent”). One item was to self-report one’s health status and the other was to compare one’s health status with others among the same age [10].

### 2.3. Design and Analysis

Our analysis was based on partial least squares structural equation modeling (PLS-SEM) [19,20] and was conducted using SmartPLS 2.0 (SmartPLS GmbH, Hamburg, Germany) [21]. According to Pirouz [22], PLS can be used for exploratory and confirmatory analyses. PLS benefits from (1) being distribution-free, (2) requiring only a small sample size, (3) an ability to process multiple dependent and independent variables simultaneously, (4) an ability to handle collinearity, and (5) an ability to process both formative or reflective indicators. Our study aimed to identify the relationships between EA, EH, PDH, GDH, BMI, and SRH. Therefore, PLS-SEM was used to test the relationships in the study.

## 3. Results

### 3.1. Descriptive Statistics

The sample consisted of 50.8% male and 49.2% female. Average value of BMI equaled 23.15 (SD = 4.66), which indicated a wide range of BMI variation among participants. The average value of EA equaled 3.81 (SD = 0.69), which indicated a positive attitude toward exercise. The average value of EH equaled 3.07 (SD = 0.95), which indicated the regular exercise habits of participants. The average value of PDH equaled 2.96 (SD = 0.76), which indicated the regularity of college students’ poor dietary habits . The average value of GDH equaled 3.34 (SD = 0.74), which indicated that the frequency of GDH’s intake was between once a week and once a day. The average value of SRH equaled 3.32 (SD = 0.75), which indicated that the health status was between good and very good.

### 3.2. Measurement Model

The factor loadings from the final PLS measurement models are all greater than 0.70 on their respective factors, which was an indication of indicator reliability. Composite reliability (ρ_c_) [23] and Cronbach’s alpha values for all scales exceeded the minimum threshold level of 0.70 [24], thus indicating the reliability of all scales used in this study (Table 1). Results revealed that the average variance extracted (AVE) for all factors exceeded the minimum threshold value of 0.50, which was an indication of the convergent validity of all scales (Table 1). Fornell’s and Larcker’s test [25] for discriminant validity revealed relatively high variances extracted for each factor compared to the interscale correlations, which was an indication of the discriminant validity of the six constructs. Secondly, we evaluated the general adjustment indicator of the model. The goodness of fit (GoF) index is fundamentally an index that measures the projection and reliability of the measurement model. Specifically, it can be understood as the geometric mean of the average communality and the average R^2^ of the endogenous latent variables [26,27]. Wetzels et al. [26] suggest that the value 0.36 is adequate for the areas of the social and behavioral sciences. Thus, doing this calculation with that value, we obtained 0.44, indicating that the model had an adequate adjustment. Lastly, we evaluated the model fit of the PLS model, with the SRMR (Standardized Root Mean Square Residual) being 0.07 and the NFI (Normed fit index) being 0.94, indicating the adequacy of the model [23].

### 3.3. Structural Model

**Figure 1:**
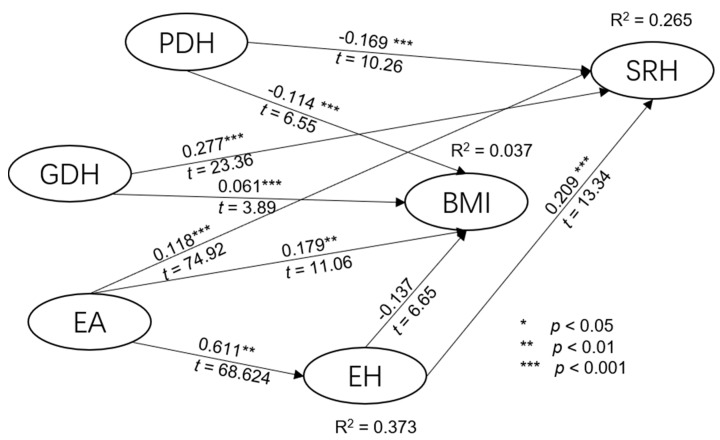
Structural model. BMI = body mass index; SRH = self-rated health; PDH = poor dietary habits; EA = exercise attitude; GDH = good dietary habits; EH = exercise habits.

The path coefficients in Table 2 and Figure 1 answer the following questions: (1) Are the dietary behavior variables directly related to SRH? The answer is yes to both good and poor dietary habits. GDH is positively related to SRH (β = 0.277, *p* < 0.001) and PDH is negatively related to SRH (β = −0.169, *p* < 0.001). Effect size and predictive relevance all indicated both GDH and PDH had predictive power on SRH. (2) Are the exercise attitude variables directly related to exercise habits? The answer is exercise attitude had significant predictive power on exercise habits (β = 0.611, *p* < 0.001 and R^2^ = 0.337). (3) Are the health behavior variables directly related to BMI? PDH is negatively related to BMI (β = −0.114, *p* < 0.001), and GDH was related to BMI (β = 0.061, *p* < 0.001). (4) Are the exercise habit variables directly related to BMI? The answer is yes. Exercise habit was related to BMI (β = −0.137, *p* < 0.001). (5) Is BMI directly related to SRH? The answer is yes. BMI is related to SRH (β = −0.136, *p* < 0.001). BMI had predictive power on SRH with *f*^2^ > 0.02 and *q*^2^ > 0.02. (6) Does BMI mediate the relation of health behavior to SRH? The answer is no since the R^2^ from those predicting constructs was extremely small. Hence, BMI does not act as a mediator among them. (7) Do exercise habits mediate the relation of exercise attitude to SRH? The answer is yes, since the path coefficient from EH to SRH was statistically significant. Hence, EH acts as a mediator among them.

Table 3 presents the direct and indirect effects and R^2^ of predictors to EH, BMI, and SRH. Exercise attitude predicted 37.5% of exercise habits while PDH, GDH, EA, and EH had little predictive power on BMI (R^2^ = 0.037). As for SRH, PDH and BMI were negatively related to SRH, while GDH, EA, and EH were all positively related to SRH. Therefore, we conclude that college students were aware of the impact of the dietary habits, exercise attitude, and exercise habits on SRH.

## 4. Discussion

This study examined the relationships of the relative contribution of dietary habits (DH), exercise attitude (EA), exercise habits (EH), and body mass index (BMI) to self-rated health (SRH) among college students. The results found EH, DH, and BMI explained 26.5% of SRH (R^2^ = 0.265), which is consistent with the study found in Sweden [28], in which the authors suggested that the levels of exercise and total physical activity were significantly associated with SRH. Taking a closer look at the path coefficients of the predictive variables, we can see that the PDH and BMI values are all negative, indicating that poor dietary habits and being overweight led to bad health status. On the other hand, GDH, EA, and EH are positively related to SRH, indicating that college students with good dietary habits and exercise habits were in better SRH status. As for the relationship of PDH, GDH, EH, and BMI, the test showed little predictive power of PDH, GDH, and EH on BMI. Finally, the relationship between EA and EH is highly correlated with an R^2^ of 0.375, indicating college students with high exercise attitude perception would also have good exercise habits, which is consistent with Dishman, Sallis, and Orenstein [29].

## 5. Conclusions

This study found that dietary habits, exercise habits, and BMI all contributed to self-rated health. Test results indicated that poor dietary habits and being overweight led to bad health status. On the other hand, good dietary habits and good exercise habits led to better health status. Among those variables, PDH, GDH, EH, and BMI all had significant positive contributions to SRH. Therefore, knowledge of healthy diet, promotion of regular exercises, refraining from poor dietary habits, and weight control may be beneficial for college student for maintaining a healthy condition.

### Limitations and Relevance for Future Investigations

This study simply used BMI as a body composition indicator. Even though BMI can be used as a convenient indicator of obesity, body fat rate is a more accurate indicator of body composition. Therefore, the suggestion is made to use body fat rate and BMI as indicators of body composition. As for students’ majors, further investigations of students’ majors can be conducted to test if students in a health-related major may influence results.

## Figures and Tables

**Table 1:** Reliability, convergent, and discriminant validity of measurement model.

Construct	Correlations					CR ^b^	A ^c^	AVE ^d^
	(1)	(2)	(3)	(4)	(5)			
(1) SRH	0.92 ^a^					0.915	0.810	0.84
(2) PDH	−0.15	0.73				0.847	0.825	0.53
(3) EA	0.29	−0.11	0.83			0.971	0.968	0.69
(4) GDH	0.38	0.05	0.18	0.77		0.858	0.780	0.60
(5) EH	0.40	−0.01	0.61	0.43	0.82	0.895	0.840	0.68

Note: SRH = self-rated health; PDH = poor dietary habits; EA = exercise attitude; GDH = good dietary habits; EH = exercise habits; ^a^ square root of AVE. ^b^ Composite reliability *(ρ_c_*) = (Σ *λ_i_*)^2^/[(Σ *λ_i_*)^2^ + Σ *Var* (*ε_i_*)], where λ_i_ is the outer factor loading, and *Var* (*ε_i_*) = 1 − *λ_i_*. is the measurement error or the error variance associated with the individual indicator variable(s) for that given factor [23]. ^c^ α = Cronbach Alpha [22]. ^d^ Average variance extracted (AVE) = (Σ *λ^2^_i_*)/[(Σ *λ^2^_i_*) + Σ *Var* (*ε_i_*)], where λ_i_ is the outer factor loading, and *Var* (*ε_i_*) = 1 − *λ_i_*, is the measurement error or the error variance associated with the individual indicator variable(s) for that given factor [23].

**Table 2:** Path coefficients of structural model.

Path	β	*t*	LL95% CI	UL95% CI	*f*^2^	*q*^2^
EA->EH	0.611 ***	32.111	0.57	0.65	0.596	0.302
PDH->BMI	−0.129 ***	3.477	−0.21	−0.05	0.017	0.008
GDH->BMI	0.035	1.202	−0.03	−0.01	0.002	0.000
EH->BMI	−0.021	0.602	−0.10	−0.06	0.000	0.000
PDH->SRH	−0.178 ***	3.477	−0.25	−0.11	0.042	0.027
GDH->SRH	0.265 ***	9.715	0.21	0.32	0.077	0.052
BMI->SRH	−0.136 ***	3.763	−0.20	−0.07	0.024	0.090
EH->SRH	0.286 ***	11.684	0.23	0.34	0.090	0.022

Note: BMI = body mass index; SRH = self-rated health; PDH = poor dietary habits; EA = exercise attitude; GDH = good dietary habits; EH = exercise habits. *t* values and CIs are calculated through bootstrapping routine with 238 cases and 5000 samples; *f*^2^: effect size; *q*^2^: predictive relevance; LL: lower level; UL: upper level; CI: confidence interval; *** *p* < 0.001.

**Table 3:** Direct and indirect effects.

Dependent Variable	Predicting Variable	Direct	Indirect	Total	R^2^
EH	EA	0.612	-	0.612	0.375
BMI	PDH	−0.114	-	−0.114	0.037
	GDH	0.061	-	0.061	
	EH	−0.137	-	−0.137	
	EA	0.179	−0.084	0.09	
SRH	PDH	−0.169	0.016	−0.153	0.265
	GDH	0.277	−0.008	0.268	
	BMI	−0.14	-	−0.14	
	EH	0.209	0.019	0.228	
	EA	0.118	−0.025	0.093	

Note: BMI = body mass index; SRH = self-rated health; PDH = poor dietary habits; EA = exercise attitude; GDH = good dietary habits; EH = exercise habits.
